# Diverse functions of clusterin promote and protect against the development of pulmonary fibrosis

**DOI:** 10.1038/s41598-018-20316-1

**Published:** 2018-01-30

**Authors:** Lizzy Peix, Iona C. Evans, David R. Pearce, Juliet K. Simpson, Toby M. Maher, Robin J. McAnulty

**Affiliations:** 1 UCL Respiratory Centre for Inflammation and Tissue Repair, Rayne Building, University College London, London, WC1E 6JF UK; 2 NIHR Respiratory Biomedical Research Unit, Royal Brompton Hospital, London, UK; 3 Fibrosis Research Group, National Heart and Lung Institute, Imperial College, London, UK; 4 Present Address: GlaxoSmithKline, Stevenage, UK; 5 Present Address: UCL Institute for Woman’s Health, University College London, London, UK

## Abstract

Pulmonary fibrosis is a progressive scarring disorder of the lung with dismal prognosis and no curative therapy. Clusterin, an extracellular chaperone and regulator of cell functions, is reduced in bronchoalveolar lavage fluid of patients with pulmonary fibrosis. However, its distribution and role in normal and fibrotic human lung are incompletely characterized. Immunohistochemical localization of clusterin revealed strong staining associated with fibroblasts in control lung and morphologically normal areas of fibrotic lung but weak or undetectable staining in fibrotic regions and particularly fibroblastic foci. Clusterin also co-localized with elastin in vessel walls and additionally with amorphous elastin deposits in fibrotic lung. Analysis of primary lung fibroblast isolates *in vitro* confirmed the down-regulation of clusterin expression in fibrotic compared with control lung fibroblasts and further demonstrated that TGF-β_1_ is capable of down-regulating fibroblast clusterin expression. shRNA-mediated down-regulation of clusterin did not affect TGF-β_1_-induced fibroblast-myofibroblast differentiation but inhibited fibroblast proliferative responses and sensitized to apoptosis. Down-regulation of clusterin in fibrotic lung fibroblasts at least partly due to increased TGF-β_1_ may therefore represent an appropriate but insufficient response to limit fibroproliferation. Reduced expression of clusterin in the lung may also limit its extracellular chaperoning activity contributing to dysregulated deposition of extracellular matrix proteins.

## Introduction

Pulmonary fibrosis is a progressive and ultimately fatal condition that currently affects more than 5 million people worldwide, the incidence is increasing and there is no known cure^1–3^. It occurs in association with several lung diseases, either in isolation, as in idiopathic pulmonary fibrosis (IPF) or in multi-organ connective tissue diseases such as systemic sclerosis (SSc)^4–6^. In the most common and aggressive form, IPF, median survival following diagnosis is less than 3 years, which is worse than for many cancers^7^. Two recently approved treatments, pirfenidone and nintedanib, slow disease progression^8,9^ but are only indicated for a small proportion of patients, have modest beneficial effects and considerable side-effects. There therefore remains a significant unmet clinical need and a requirement to further characterize the pathogenesis of pulmonary fibrosis and develop more effective treatments.

The development of pulmonary fibrosis is incompletely understood, although aberrant injury repair mechanisms, with persistence of increased numbers of fibroblast/myofibroblast cells driving excess production of extracellular matrix proteins, are considered to be central to its pathogenesis^10^. Clusterin (also called apolipoprotein J) is a heterodimeric secretory glycoprotein, ubiquitously expressed in human tissues and body fluids. In a proteomic analysis of bronchoalveolar lavage fluid (BALF) Kim and co-workers showed that clusterin levels were approximately 7-fold lower in IPF compared with controls^11^. In addition, studies of fibrosis in other organs, including heart, kidney, liver, and in animal models suggest that down- or up-regulation of clusterin enhance or limit the development of fibrosis respectively^12–15^ suggesting that clusterin may play an important role in the pathogenesis of fibrosis. Clusterin is a multifunctional protein. It is known to be involved in the regulation of proliferation, differentiation and survival of cells including epithelial cells, smooth muscle cells and synoviocytes^16–22^. Furthermore, TGF-β_1_, a potent pro-fibrotic mediator, has been reported to up-regulate clusterin expression in epithelial cells affecting differentiation and apoptosis^23–26^. In addition, clusterin acts as an extracellular chaperone involved in promoting the appropriate folding and conformation of extracellular proteins and shielding proteins under conditions of tissue stress^27,28^. However, the localization of clusterin in normal and fibrotic lung, the mechanisms contributing to its down-regulation in IPF BALF and its role in the pathogenesis of pulmonary fibrosis have not been investigated.

In this study we immunohistochemically examined the expression and localization of clusterin in normal and fibrotic human lung. Based on the characteristic pattern of staining and differences observed in fibrotic lung we investigated its extracellular protein binding characteristics and the functional effects of clusterin on human lung fibroblast proliferation, differentiation, collagen synthesis and apoptosis *in vitro* in cells isolated from control and fibrotic lung. Potential mechanisms for the regulation of clusterin and its role in mediating the pro-fibrotic effects of TGFβ_1_ were investigated in control lung fibroblasts and compared to its effects on fibrotic fibroblasts. These data provide novel insights into the functional role of clusterin, the mechanisms by which it is regulated in fibroblasts in the human lung and further contribute to our understanding of the pathogenesis of pulmonary fibrosis.

## Results

### Immunohistochemical Localization of Clusterin in Normal and IPF Lung

**Figure 1:**
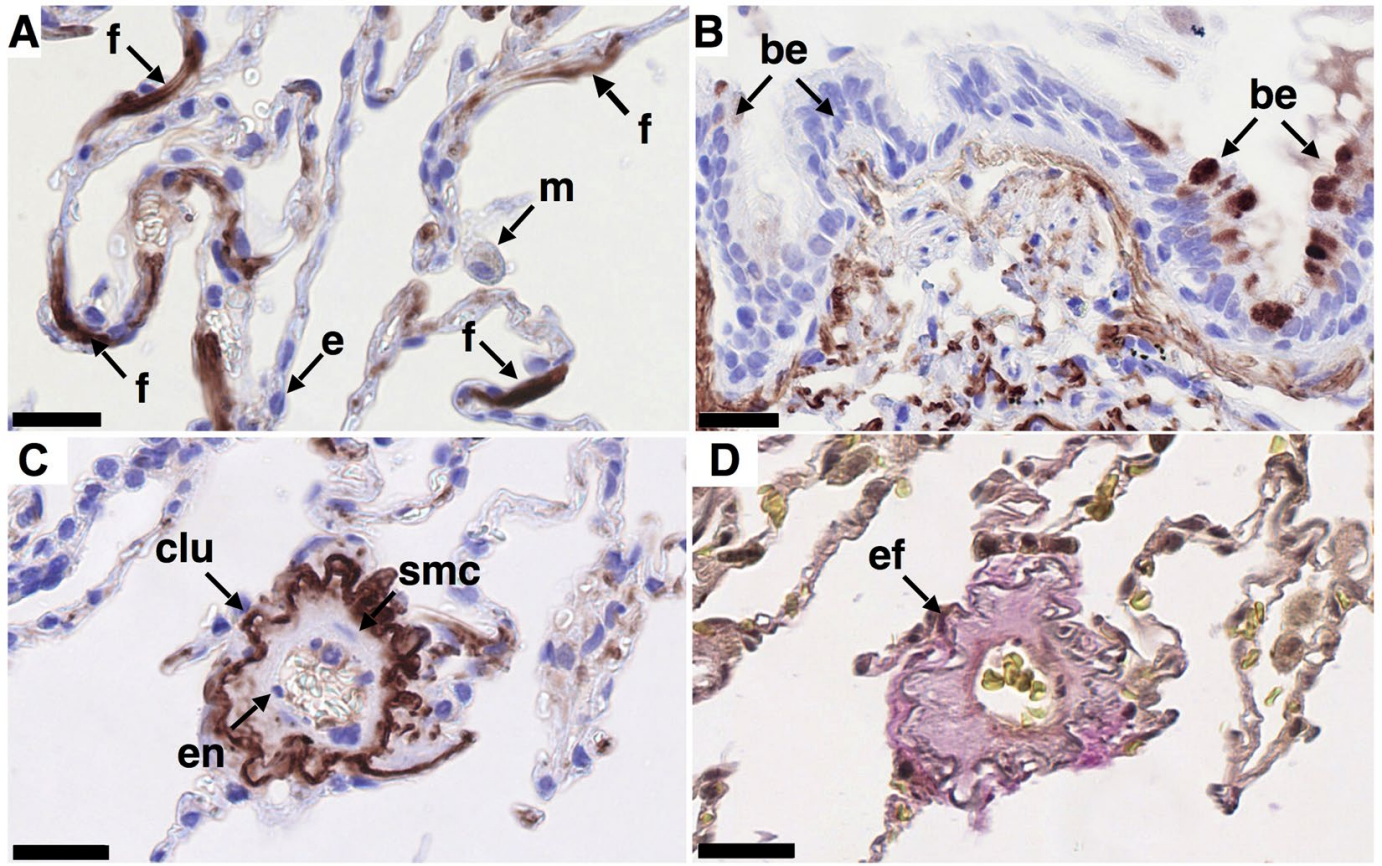
Localization of clusterin in normal human lung. Clusterin was detected immunohistochemically by staining formalin-fixed, paraffin embedded 3 μm sections of human control lung tissue. Representative images of clusterin (clu, A-C, brown/red, nuclei - blue) and elastic fibers ((**D**), grey/black) in tissue obtained from control lung (n = 3). Clusterin localizes to fibroblast-like cells (**A**), to small areas of bronchial epithelial cells (**B**) and to elastic fibers in blood vessels and alveolar walls (**C**,**D**) serial sections). Clusterin was not detectable in macrophages, alveolar epithelial cells (**A**) or endothelial cells (**C**). Different cell populations/structures are indicated by arrows: f - fibroblast-like cell, m - macrophage, e – alveolar epithelial cell, be - bronchial epithelial cell, en - endothelial cell, smc – smooth muscle cell, ef - elastic fibers. Scale bar represents 25 µm.

In normal human lung, clusterin was localized to fibroblast-like cells (Fig. 1A) and sporadic areas of bronchial epithelial cells (Fig. 1B). Additional clusterin staining was observed in vessel walls (Fig. 1C), where it co-localized with elastin as assessed by Elastica van Gieson (EvG) staining (Fig. 1D). Staining for clusterin in macrophages was weak (Fig. 1A) or undetectable. Clusterin was also undetectable in alveolar epithelial, endothelial and smooth muscle cells (Fig. 1A and C).

**Figure 2:**
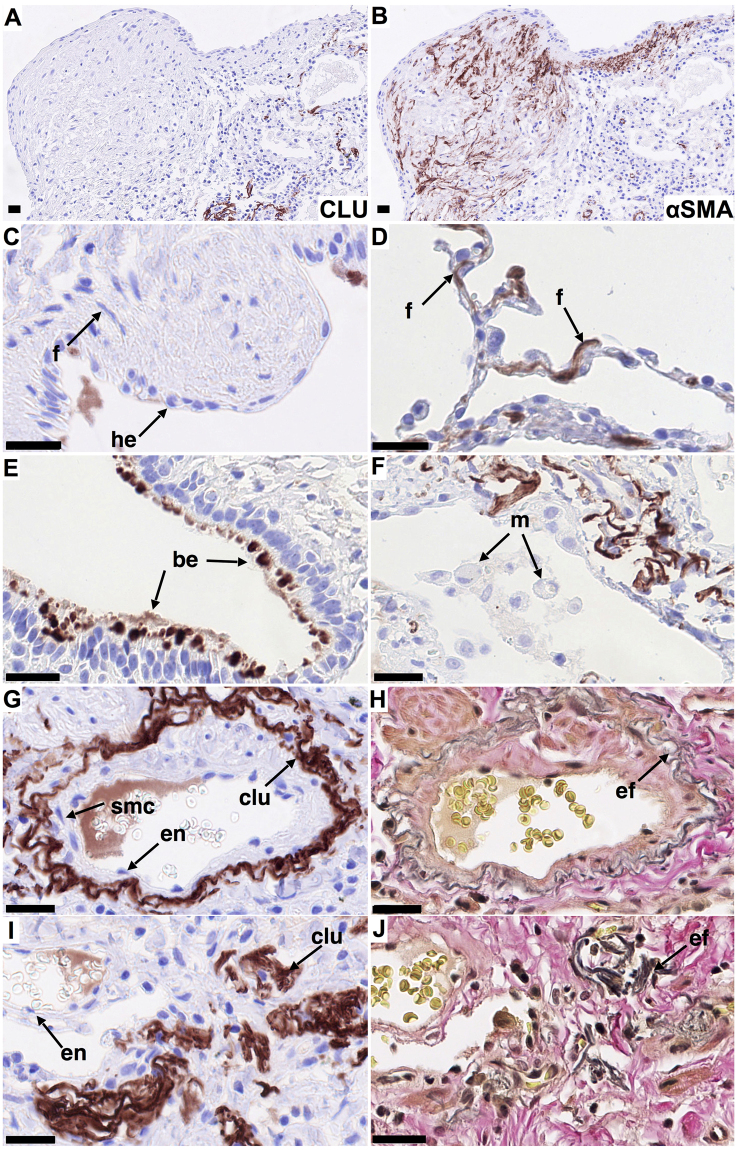
Localization of clusterin in IPF lung. Immunohistochemical staining for clusterin was performed on formalin-fixed, paraffin embedded 3 μm sections of IPF lung tissue (n = 3). Clusterin staining (clu, A, C-G, I brown/red, nuclei - blue) and staining for elastin (H, J, EvG, grey/black) in representative tissue sections. Clusterin is undetectable in αSMA positive myofibroblasts (**A,B**), forming and in cells overlying fibroblastic foci (**A,C**), compared to strong staining of fibroblast-like cells in morphologically normal non-fibrotic areas (**D**). Clusterin was observed sporadically in bronchial epithelial cells but more frequently than in controls (**E**). Similar to control lung, clusterin colocalized with elastin (**G,H**) and was undetectable in macrophages, smooth muscle and endothelial cells (**F,G**). Clusterin also colocalized with amorphous elastin aggregates in dense fibrotic regions (**I,J**). Different cell populations/structures are indicated by arrows; f - fibroblast-like cell, m - macrophage, he - hyperplastic epithelial cell, be - bronchial epithelial cell, en – endothelial cell, smc – smooth muscle cell, ef- elastic fibers. Scale bar represents 25 µm (**A–J**).

In contrast, in IPF lung clusterin staining was weak or undetectable in fibroblasts associated with fibrotic regions (Fig. 2A,C) and particularly in αSMA positive myofibroblasts in fibroblastic foci (Fig. 2A,B), whereas fibroblasts in morphologically normal areas of IPF-lung showed strong clusterin staining comparable to that of fibroblast-like cells in control lungs (Fig. 2D). Hyperplastic epithelial cells overlying fibroblastic foci showed weak or no clusterin staining (Fig. 2A,C). Clusterin staining of bronchial epithelial cells was sporadic but more extensive in IPF lungs compared with controls (Fig. 2E). Furthermore, IPF lungs showed strong clusterin staining associated with elastin in vessel walls (Fig. 2G; EvG staining H), but also with amorphous elastin rich deposits in fibrotic areas (Fig. 2I; EvG staining J). Clusterin staining of macrophages, smooth muscle cells and endothelial cells of IPF lungs was generally weak or undetectable as in control lungs (Fig. 2F,G).

### Clusterin Expression in Control and Fibrotic Lung Fibroblasts and its Regulation by TGF-β_1_

**Figure 3:**
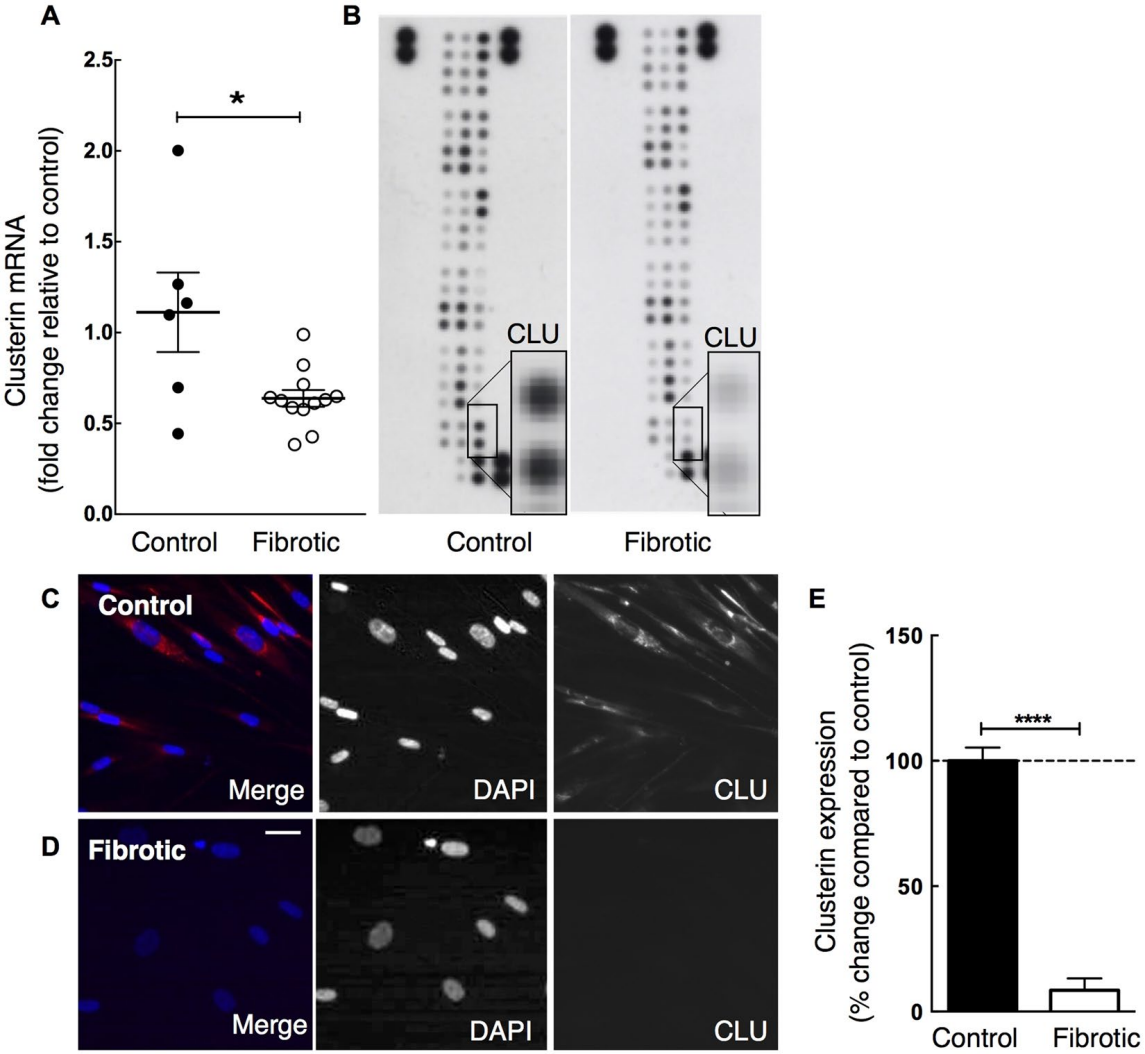
Clusterin gene expression and protein levels are decreased in fibrotic compared with control lung fibroblasts. Fibroblasts isolated from human control and fibrotic lung were grown in monolayer culture and clusterin mRNA and protein levels were detected via microarray, proteome profiler and immunofluoresecence analysis. (**A**) Microarray analysis of mRNA shows decreased clusterin gene expression in fibroblasts derived from fibrotic lungs (open circles; n = 5 IPF and 7 SSc) compared with controls (closed circles; n = 6). Proteome profiler array analysis (**B**, clusterin - CLU) and immunofluorescence staining of control and fibrotic lung fibroblasts (**C,D**) confirms low clusterin protein expression in fibrotic compared with control fibroblasts *in vitr*o. (**E**) Semi-quantitative analysis of clusterin staining (**C,D**), clusterin signal (pixel intensity) normalized to cell numbers per visual field (n = 6). Data is representative of three individual experiments. *P < 0.05, ****P < 0.0001; Scale bar in D represents 10 µm.

To confirm the changes in clusterin observed in IPF lung we examined the expression of clusterin in fibroblasts isolated from control and fibrotic lung. Although IPF and SSc have different aetiologies both result in pulmonary fibrosis, which involves a TGF-β1 - mediated component. We were therefore interested in clusterin mRNA levels in fibroblasts derived from both IPF and SSc patients. Consistent with the immunohistochemical data, clusterin mRNA expression was reduced in fibrotic (IPF and SSc derived) compared with control lung fibroblasts (Fig. 3A), suggesting that this change in clusterin messenger may be fibrosis related. The differences were confirmed at protein level in representative donor fibroblast isolates assessed by protein array analysis (Fig. 3B) and immunofluorescent staining (Fig. 3C–E).

**Figure 4:**
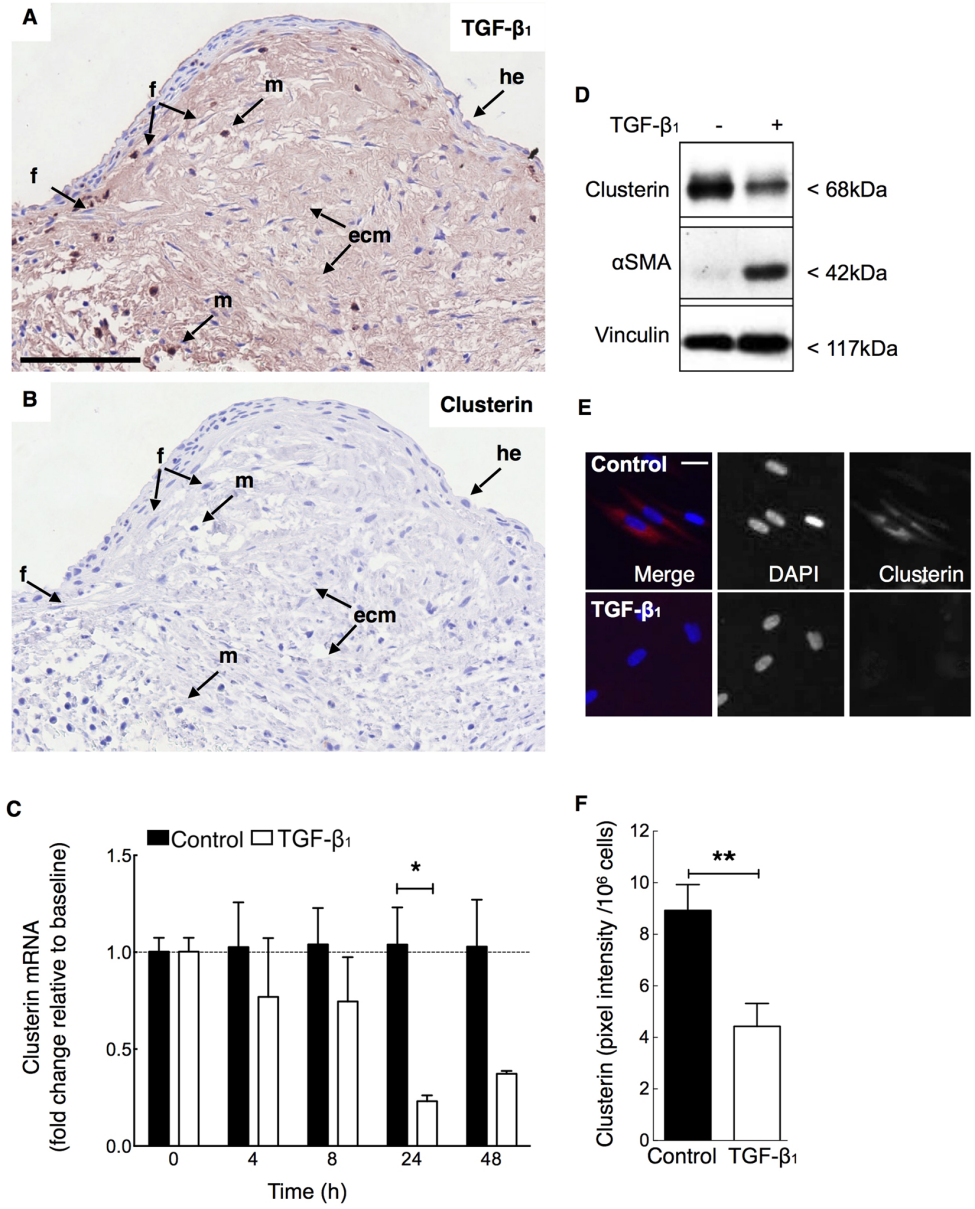
TGF-β_1_ associates with areas of decreased clusterin expression in fibrotic lung and down-regulates fibroblast clusterin mRNA and protein expression *in vitro*. Serial sections prepared from IPF lung (n = 3) were stained immunohistochemically to localize TGF-β_1_ staining ((**A**), red/brown, nuclei blue) and clusterin ((**B**), red/brown, nuclei blue) in fibroblastic foci. (**A,B**) Representative images of immunohistochemical staining suggest that TGF-β_1_ localizes to ECM, fibroblasts and macrophages, whilst staining for clusterin is weak or undetectable. (**C**) *In vitro* analysis of clusterin mRNA levels in TGF-β_1_ stimulated (40 pM) human lung fibroblasts was performed via qRT-PCR and shows a time-dependent down-regulation of clusterin expression that was maximal at 24–48 h (n = 3) in response to TGF-β_1_. Clusterin protein levels were also down-regulated in response to TGF-β_1_ (40 pM) compared to control at 24 h and 48 h as demonstrated by western blotting at 24 h (**D**) and immunofluorescent staining at 48 h (E, red, nuclei - blue). (**F**) Semi-quantitative analysis of fluorescent signal of panel E: clusterin signal (pixel intensity) was normalized to cell numbers per visual field and compared to control (n = 6). Full-length western blots are presented in Supplementary Figure e4. Different cell populations/structures are indicated by arrows; f - fibroblast-like cell, m - macrophage, he - hyperplastic epithelial cell, ecm – extracellular matrix. *P < 0.05, **P < 0.01; scale bar 100 µm (A) and 10 µm (**E**).

**Figure 5:**
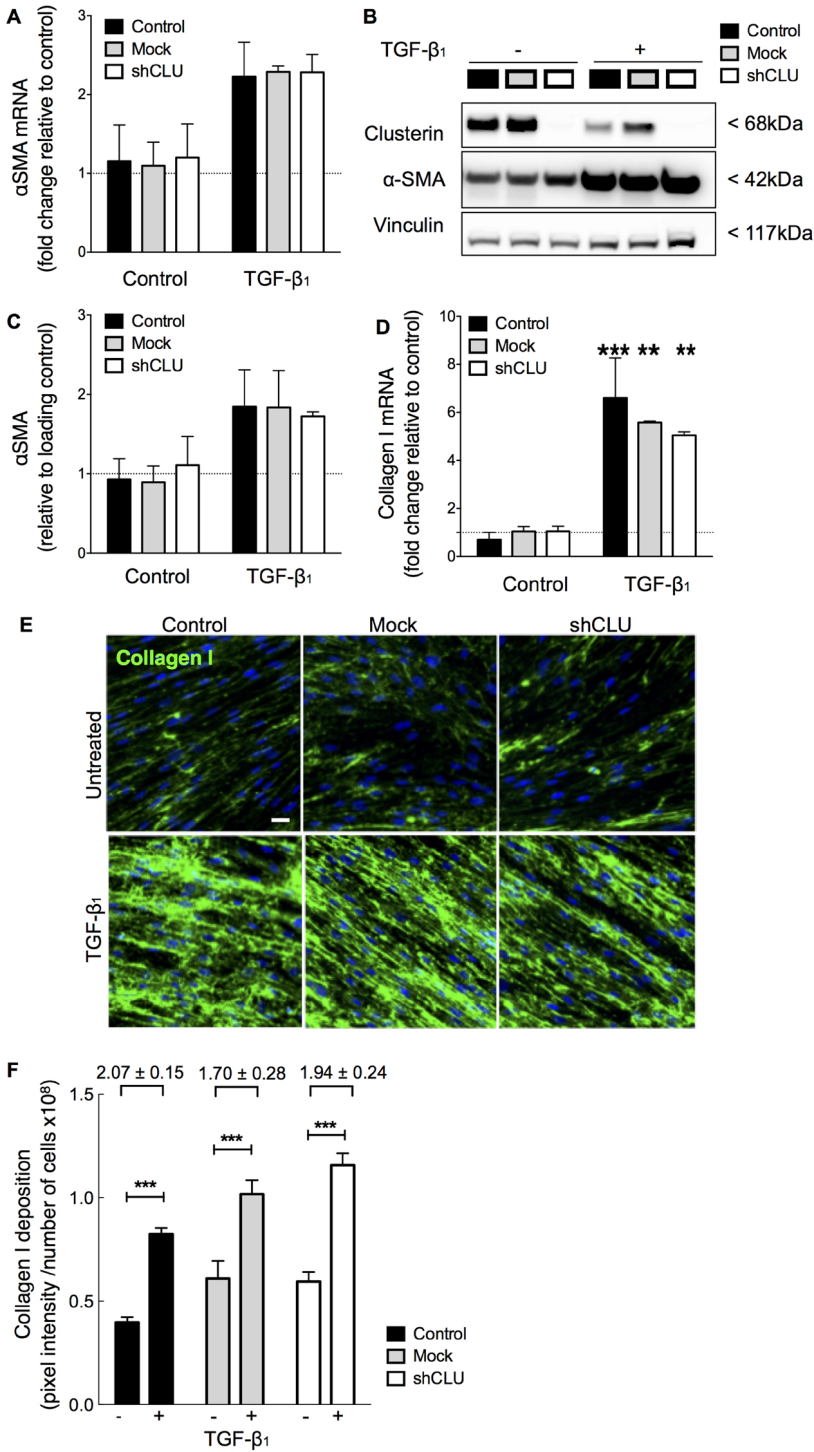
Effect of clusterin deficiency on TGF-β_1_-induced myofibroblast differentiation and collagen deposition. Lung fibroblasts were transduced with clusterin shRNA (shCLU, open bars) and mock shRNA vectors (grey bars) or remained untransfected (black bars). αSMA mRNA was assessed via qRT-PCR (**A**) and clusterin and αSMA protein levels detected by western blotting (**B,C** quantification). 48 h following TGF-β_1_ stimulation (40 pM) αSMA mRNA and protein were increased. Basal and increased levels of αSMA mRNA and protein, however, did not vary between clusterin deficient, mock-transduced and control fibroblasts. Collagen mRNA (**D**) and deposition (**E**,**F** quantification) assessed by qRT-PCR and immunofluorescence staining were significantly increased in response to TGF-β_1_. Although, basal and TGF-β_1_ induced changes in collagen levels varied between clusterin deficient, mock and control fibroblasts, the overall fold-increase in collagen mRNA and deposition levels did not significantly change between clusterin deficient fibroblasts and control/mock fibroblasts. Data generated in A-F is representative of two individual experiments with fibroblasts derived from 2 donors. Full-length western blots are presented in Supplementary Figure e5. Scale bar in E represents 10 µm. **P < 0.01, ***P < 0.001 compared with untreated controls respectively.

Whilst previous studies suggest TGF-β_1_ up-regulates clusterin expression in epithelial cells^24^, there is little information on its effect on fibroblasts. TGF-β_1_ is a major pro-fibrotic mediator that induces fibroblast to myofibroblast differentiation with up-regulation of αSMA and collagen expression. Since fibroblastic foci are rich in extracellular matrix-bound TGF-β_1_ (Fig. 4A)^29,30^ and myofibroblasts associated with these foci exhibit low clusterin expression (Figs 2A,C and 4B), we hypothesized that TGF-β_1_ may regulate fibroblast/myofibroblast clusterin expression. We therefore examined the effect of TGF-β_1_ on clusterin expression in fibroblasts *in vitro*: Stimulation of control lung fibroblasts with TGF-β_1_ reduced clusterin mRNA levels in a time-dependent manner (Fig. 4C). Protein analysis by western blot (Figs 4D, 5B) and immunofluorescence analysis (Fig. 4E,F) post TGF-β_1_ stimulation, confirmed the down-regulation of clusterin by TGF-β_1_.

### Effect of Low Clusterin Expression on Myofibroblast Differentiation in Control Lung Fibroblasts

To assess the importance of clusterin in regulating TGF-β_1_-induced myofibroblast differentiation, control fibroblasts were transduced with lentiviral shRNA targeting the clusterin gene. To exclude effects of transduction-induced changes, mock non-silencing shRNA-transduced fibroblasts were compared with untransduced controls. shRNA-induced clusterin knockdown compared with both mock-transduced and control was confirmed for mRNA, protein, and secretory clusterin (see Supplementary Figure e1).

In order to address whether clusterin deficiency affects basal and TGF-β_1_-induced αSMA, collagen mRNA and protein deposition, non-transduced control, mock-transduced and shRNA transduced clusterin-deficient fibroblasts were treated with or without TGF-β_1_ (Fig. 5). Although TGF-β_1_ induced αSMA mRNA and protein as assessed by qRT-PCR and Western blot analysis (Fig. 5A–C), basal and induced αSMA levels did not show significant differences between control, mock and shCLU transduced fibroblasts. As expected, TGF-β_1_ significantly induced collagen I mRNA and protein deposition as assessed by qRT-PCR (Fig. 5D) and immunofluorescent staining (Fig. 5E–F), respectively. However, there was no significant difference in response to TGF-β_1_ between control, mock transduced and shCLU fibroblasts. This suggests that the observed TGF-β_1_ induced down-regulation of clusterin is independent of its effect on myofibroblast differentiation, collagen synthesis, and deposition. We therefore investigated whether clusterin deficiency functionally affects other important biological processes in lung fibroblasts, including proliferation, migration, and cell survival.

### Effect of Clusterin Deficiency on Fibroblast Proliferation

**Figure 6:**
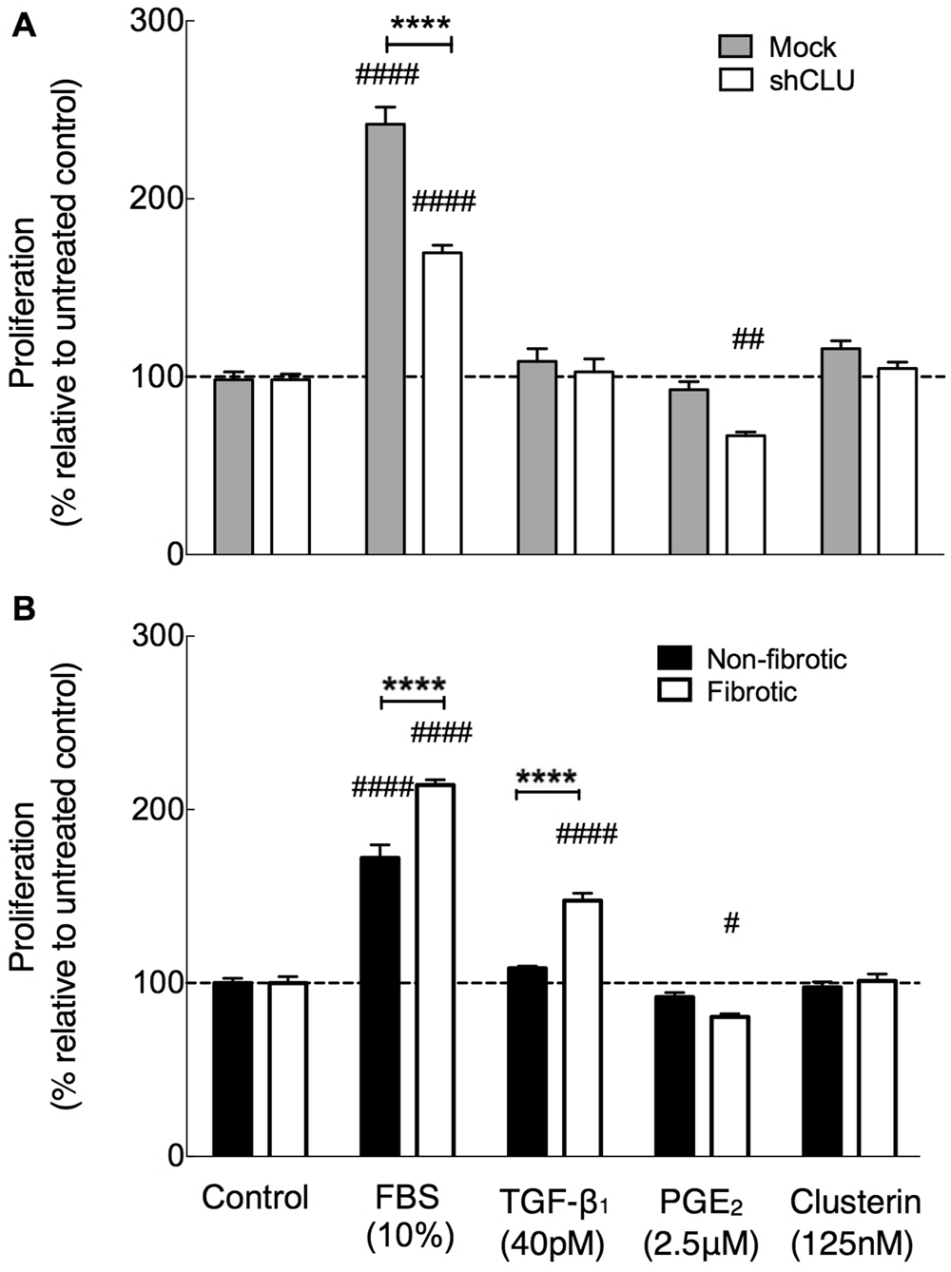
Effect of clusterin deficiency on lung fibroblast proliferation.Lung fibroblasts were transduced with clusterin shRNA (shCLU, open bars) and mock shRNA vectors (grey bars) or remained untreated (black bars). Proliferation in shRNA-mediated clusterin deficient fibroblasts (**A**) or fibrotic lung fibroblasts (**B**) compared with controls was assessed in response to the indicated stimuli for 48 h or 72 h for FBS by counting DAPI-positive nuclei in a high-throughput immunofluorescence assay. Cell numbers were normalized to cell counts of controls (0.4% FBS in DMEM) and expressed as percent change in proliferation relative to control (n = 6). Data representatives of two individual experiments with fibroblasts derived from 1 donor per group. Significances compared with controls are marked with (#) symbol and significances between controls vs. shCLU or non-fibrotic vs. fibrotic lung fibroblasts are indicated with (*). ^*/#^P < 0.05, ^**/##^P < 0.01, ***/^###^P < 0.001, ****/^####^P < 0.0001.

Previous studies have indicated that clusterin promotes proliferation of renal tubular epithelial and vascular smooth muscle cells after injury^20,31^. In order to examine the effects of reduced clusterin expression on lung fibroblast proliferation, shRNA-mediated clusterin-deficient and control (mock-transduced) fibroblasts were stimulated with serum, TGF-β_1_ or PGE_2_ (Fig. 6A). Serum significantly increased proliferation of control and clusterin deficient fibroblasts. However, the response to serum-induced proliferation was diminished by approximately 50% in clusterin-deficient fibroblasts compared with controls (Fig. 6A). TGF-β_1_ did not affect the proliferative response in control or clusterin-deficient fibroblasts and PGE_2_ significantly reduced proliferation in clusterin-deficient fibroblasts compared with control. Interestingly, exogenous clusterin did not affect fibroblast proliferation in control or clusterin-deficient fibroblasts (Fig. 6A). Together, these data suggest that endogenously generated intracellular clusterin but not exogenous clusterin is involved in promoting fibroblast proliferation. The response to the same mediators was also compared between representative control and fibrotic lung fibroblasts, which express low levels of clusterin (Fig. 6B). Serum induced proliferation in non-fibrotic and fibrotic lung fibroblasts. However, in contrast to shRNA-mediated clusterin deficient fibroblasts, naturally clusterin-deficient fibrotic lung fibroblasts exhibited a greater proliferative response than controls. This difference may be associated with changes in fibrotic lung fibroblasts unrelated to diminished clusterin expression. Moreover, fibrotic lung fibroblasts displayed a proliferative response to TGF-β_1_ compared with untreated and non-fibrotic controls. Consistent with shRNA-mediated clusterin deficiency, PGE_2,_ but not exogenous clusterin, reduced the proliferation of fibrotic lung fibroblasts.

### Clusterin Protects Fibroblasts from Apoptosis

We and others have previously demonstrated that fibrotic lung fibroblasts are more resistant to FasL-induced apoptosis when compared with control fibroblasts^32,33^. Clusterin has been associated with impaired apoptosis in prostate cancer cells via interaction with activated Bax^22^. In order to determine the role of clusterin in lung fibroblast apoptosis *in vitro*, we investigated the effects of clusterin deficiency and/or exogenous clusterin on FasL - induced apoptosis in control and IPF lung fibroblasts.

**Figure 7:**
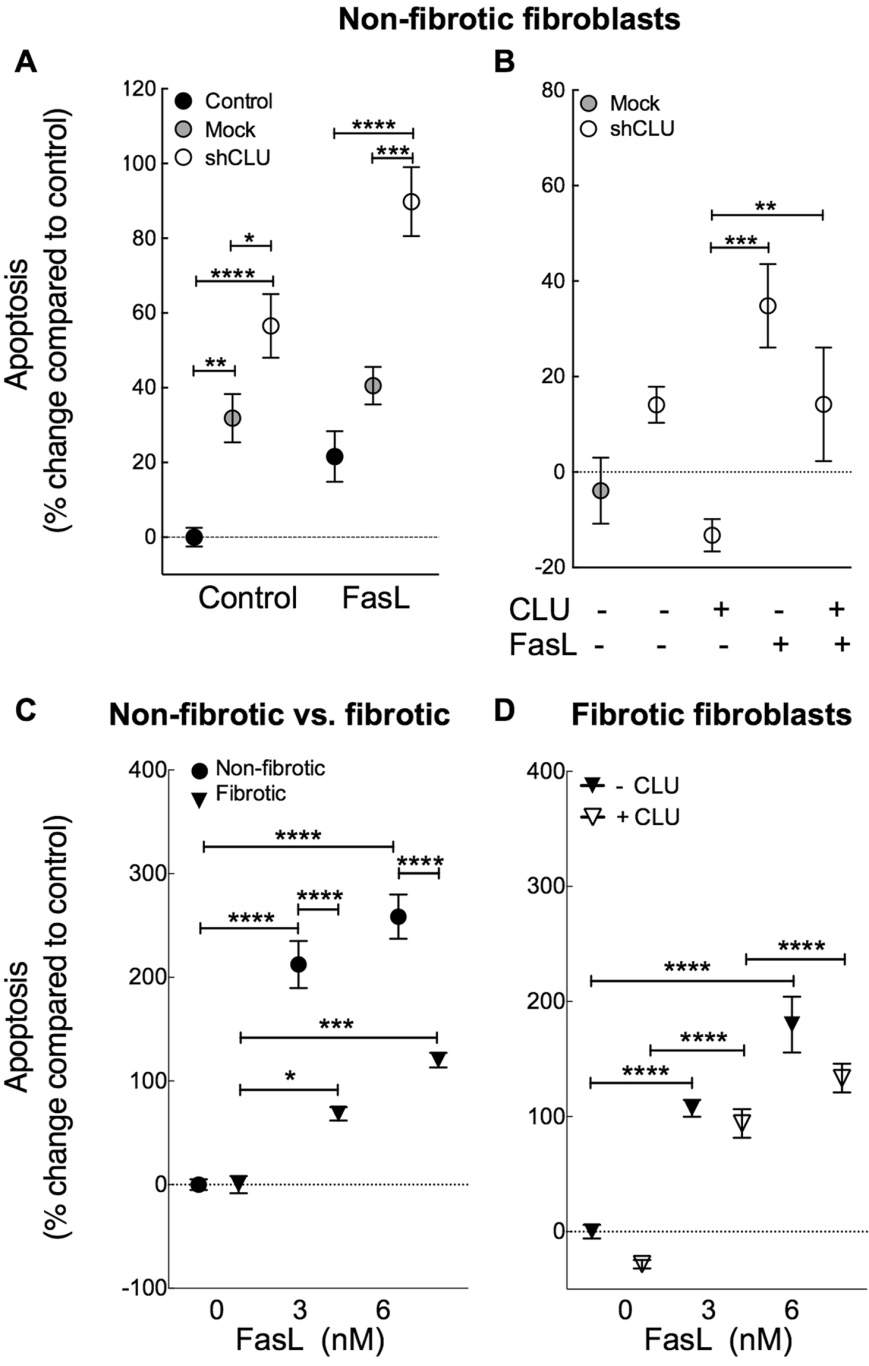
Effect of clusterin deficiency on apoptosis. Lung fibroblasts were transduced with clusterin shRNA (shCLU, open circles) and mock shRNA vectors (grey circles) or remained untransfected (black circles). Lung fibroblasts were seeded and treated with FasL (3 nM – 6 nM) and/or exogenous clusterin (CLU, 125 nM) for 19 h or remained untreated. Apoptotic cells (Annexin V+ and Annexin V+/ DAPI+ cells) were assessed via FACS analysis post staining of apoptotic cells with annexin V – Alexa647 and DAPI (mean ± SEM, n = 5). (**A**) shRNA-induced clusterin deficiency sensitized fibroblasts to basal and FasL-induced apoptosis, and this could be overcome by addition of exogenous clusterin (**B**). (**C**) Basal apoptosis in representative control (9.37 ± 0.63%) and fibrotic (11.6 ± 0.69%) lung fibroblast isolates was not significantly different. Fibrotic lung fibroblasts were more resistant to FasL-induced apoptosis compared with controls (**C**) and exogenous clusterin tends to reduce basal and FasL-induced apoptotic levels further (**D**). Data representative of at least two individual experiments with fibroblasts derived from 1 donor per group. *P < 0.05, **P < 0.01, ***P < 0.001, ****P < 0.0001.

As expected, treatment with FasL resulted in increased fibroblast apoptosis in control fibroblasts (see Fig. 7 and Supplementary Figure e2). shRNA-mediated clusterin-deficient fibroblasts exhibited higher basal rates of apoptosis and demonstrated increased sensitivity to FasL-induced apoptosis compared to non–transduced and mock-transduced controls (Fig. 7A). These effects could be overcome by administration of exogenous clusterin (Fig. 7B), suggesting that exogenous or secreted clusterin protects lung fibroblasts from apoptosis *in vitro*. Since clusterin expression is reduced in IPF lung fibroblasts compared with controls we sought to determine the effects of exogenous clusterin on apoptosis. As previously described^32^, we found that fibrotic lung fibroblasts were more resistant to FasL-induced apoptosis than controls (Fig. 7C). In accordance with the protective effect of exogenous clusterin in control fibroblasts, we found that exogenous clusterin tended to reduce basal and FasL-induced apoptosis in fibrotic lung fibroblasts but this was not statistically significant (Fig. 7D). Together, this suggests that exogenous clusterin protects fibroblasts from apoptosis.

### Clusterin Does Not Affect Fibroblast Migration

Clusterin has been demonstrated to induce chemotaxis of human monocytes, murine macrophages^34^ and porcine vascular smooth muscle cells^18^ at concentrations of 3–10 µg/ml. However, little is known about the role of clusterin in regulating migration of lung fibroblasts. Since lung fibroblasts secrete levels of clusterin within this concentration range (Supplementary Figure e1E) we examined whether clusterin deficiency or addition of exogenous clusterin could affect lung fibroblast migration in control and fibrotic lung fibroblasts. Transwell migration assays were conducted with control (mock-transduced), shRNA-mediated clusterin-deficient and fibrotic lung fibroblasts in response to PDGF-BB, TGF-β_1_ or exogenous human plasma derived clusterin. Consistent with previous reports^35–37^, PDGF-BB significantly increased fibroblast migration by approximately 3 fold compared with unstimulated controls (see Supplementary Figure e3A) while, in accordance with previous reports TGF-β_1_ did not affect chemotaxis^36^. Exogenous clusterin did not affect chemotaxis in clusterin-deficient or fibrotic lung fibroblasts compared with controls. Furthermore, basal migration in clusterin-deficient and fibrotic lung fibroblasts did not differ when compared with untreated controls or in response to PDGF-BB and TGF-β_1_ (see Supplementary Figure e3B,C). Together, these data suggest clusterin does not mediate chemotaxis of control and fibrotic lung fibroblasts *in* vitro.

## Discussion

To our knowledge this is the first demonstration of clusterin expression in normal compared with fibrotic lung. Since clusterin has previously been found to be down-regulated in BALF of individuals with IPF, we sought to determine the pattern of tissue localization in normal lung and any changes that occurred in fibrotic lung and following on from this we examined the functional consequences of altered clusterin expression in control and fibrotic lung fibroblasts. Immunohistochemical analysis provided evidence of strong staining for clusterin in fibroblasts in the alveolar septum of normal human lung. Similar staining was observed in apparently normal areas of IPF lung but in fibrotic regions of lung staining for clusterin was weak or undetectable, especially in fibroblasts/myofibroblasts in fibroblastic foci. Furthermore, weak clusterin staining was associated with areas of strong TGF-β_1_ staining and αSMA positive cells. Consistent with these observations fibroblasts/myofibroblasts derived from IPF lung expressed reduced levels of clusterin mRNA and protein compared with control lung fibroblasts and exposure of control fibroblasts to TGF-β_1_ resulted in a significant down-regulation of clusterin expression. This is in contrast to previous studies in epithelial cells in which TGF-β_1_ has been reported to up-regulate clusterin expression^24,25^.

Clusterin biology is complex, at least partly, due to the expression of alternatively spliced isoforms, their different intra- and extra-cellular localization, as well as, cell and tissue specific expression and function of the different isoforms. Previous reports postulated that alternative splicing of the clusterin mRNA results in an N-terminal truncated, non-secreted nuclear clusterin isoform in human mammary gland carcinoma cell line MCF-7^38,39^ and HEK-293 cells^39^. However, more recent studies have not confirmed this finding in other cell lines and it has been suggested by Rizzi *et al*. that an alternative transcript encoding for nuclear clusterin may be cell specific or an experimental artefact^40^. Concerted mRNA-targeted studies are required to investigate if these observations in carcinoma and embryonic kidney cell lines are true for primary lung cells, including lung fibroblasts. In the present study, clusterin localized to the cytoplasm of primary lung fibroblasts under basal conditions and in response to TGF-β_1_. As demonstrated by western blot analysis under reducing conditions clusterin at sizes of ∼ 68 kDa (full length, cytoplasmic clusterin) and single clusterin chains ∼ 36–38 kDa each, but no truncated (∼49 kDa) nuclear isoform were detectable (see Supplementary Figures e4 and e5). This was consistent with immunofluorescence staining of cells which also only detected cytoplasmic clusterin with no evidence for the presence of a nuclear localized form in lung fibroblasts (Fig. 3C).

TGF-β_1_ is a major profibrotic mediator, which drives myofibroblast differentiation and excessive collagen deposition. Furthermore, it has been shown in clusterin deficient mice that the frequency of αSMA-positive myofibroblasts were increased compared with wild-type mice^12^. We therefore investigated whether the pro-fibrotic effects of TGF-β_1_ are mediated by down-regulation of clusterin in fibroblasts. shRNA-mediated down-regulation of clusterin did not affect basal or TGF-β_1_-induced fibroblast differentiation assessed by αSMA expression, collagen I expression and deposition. This suggests that the TGF-β_1_-induced down-regulation of clusterin is independent of its effects on fibroblast to myofibroblast differentiation. This contrasts with smooth muscle cells (SMC) in which clusterin has been suggested to be involved in phenotypic modulation and differentiation^16,41^ and a reduction in clusterin expression was associated with increased αSMA expression^42^.

Furthermore, we found that shRNA-induced clusterin-deficiency decreased fibroblast proliferation in control fibroblasts in response to serum. This is in accordance with reports of clusterin promoting proliferation in VSMC (vascular smooth muscle cells) *in vitro*^31^. In contrast, fibroblasts derived from IPF lung that have reduced clusterin expression, exhibited a hyperproliferative phenotype compared with non-fibrotic control fibroblasts that express higher levels of clusterin. The reason for this is not known but is likely due to a potential dominance of clusterin-independent, pro-proliferative mechanisms in fibrotic lung fibroblasts, resulting in enhanced proliferation despite low clusterin expression. For example, the inability of fibrotic lung fibroblasts to up-regulate COX-2 and subsequently PGE_2,_ which is a potent inhibitor of fibroblast proliferation, in response to TGF-β_1_, results in the dominance of TGF-β_1_-induced PDGF expression and enhanced proliferation of fibrotic lung fibroblasts^43^. Enhanced production of PGE_2_ could also be responsible for the inhibition of proliferation in clusterin shRNA treated control fibroblasts. Alternatively, previous studies in cancer cells, suggest that effects of siRNA induced silencing or overexpression of clusterin on cell proliferation and apoptosis, are mediated via the PI3K/ AKT pathway^44,45^. Although further studies are required to characterize the mechanisms responsible for the effects of TGF-β_1_ on clusterin expression, clusterin-mediated effects on proliferation and apoptosis in lung fibroblasts, it is becoming clear that the effects of clusterin are cell and tissue specific.

In IPF several lines of evidence demonstrate that uncontrolled fibroblast accumulation is at least partly due to fibroblast/myofibroblast resistance to apoptosis^32,33^. Previous studies have demonstrated that down–regulation of clusterin in rheumatoid arthritis (RA), is associated with synovial fibroblast resistance to FasL-mediated apoptosis^46^ and that transgenic overexpression of clusterin in RA synovial fibroblasts promoted apoptosis^17^. We therefore investigated whether clusterin deficiency contributes to the resistance of fibrotic lung fibroblasts to FasL-induced apoptosis. Our *in vitro* studies showed that unlike in synovial fibroblasts, shRNA-mediated down-regulation of clusterin induced basal and FasL-induced apoptosis in control lung fibroblasts. Furthermore, we showed that the increase in basal and FasL-induced apoptosis seen with low clusterin expression could be overcome by addition of exogenous clusterin. This is consistent with previous reports in VSMC, where exogenous clusterin protected from H_2_0_2_-induced apoptosis^16^. We confirmed the previously reported resistance of fibrotic lung fibroblasts to FasL-induced apoptosis. Furthermore, we found that exogenous clusterin tended to further potentiate this resistance to apoptosis. Together, our *in vitro* studies suggest that clusterin is protective against apoptosis in normal and fibrotic lung fibroblasts *in vitro*. This is consistent with the reported effects of clusterin in VSMC but in contrast to its effects in synovial fibroblasts further supporting the notion that the effects of clusterin are cell and tissue dependent.

We found that clusterin is sporadically expressed in bronchial epithelial cells of the normal adult lung. However, in IPF lung clusterin staining of bronchial epithelial cells was both more frequent and intense. In rodents, clusterin is expressed embryologically in the lung epithelium during branching morphogenesis^47–49^ but is not expressed in post-developmental or healthy adult bronchial epithelium^48,49^. Infection and injury have been reported to induce the expression of clusterin in the bronchial epithelium^49^ and epithelium of other organs including the ileum of Crohn’s disease patients^50^ and in experimental kidney injury^12^ where clusterin is thought to be protective. The increased expression of clusterin in the bronchial epithelium in IPF may therefore be a reflection of epithelial stress/injury or, alternatively, a component of the aberrant re-expression of developmental genes that occurs in IPF^51^.

Immunolocalisation of clusterin clearly demonstrated its association with elastin in vessel walls. Staining was increased in IPF lung and was also observed in amorphous elastin-rich deposits within fibrotic regions. Elastin deposition has previously been shown to be increased in IPF lungs compared with controls^52,53^ but there is also evidence for increased levels of neutrophil elastase- and MMP7-mediated elastin degradation in IPF^54,55^. Clusterin has previously been found to associate with elastin in human photoaged skin^56^, cirrhotic liver^28^ and exfoliation syndrome^57^ but has not, as far as we are aware, previously been observed in pulmonary fibrosis. Several potential functions have been proposed for the association of clusterin with elastin. Clusterin, like small heat shock proteins, is a molecular chaperone which, through hydrophobic interactions, is able to bind and stabilize partially folded, stressed proteins and long-lived protein intermediates that slowly aggregate, including elastin, shielding and preventing their precipitation^56,58^. Alternatively, clusterin may contribute to the clearance of defective and degraded elastin via megalin/gp330 receptor-mediated endocytosis^56,59^. Further studies would be necessary to determine the precise roles of the association of clusterin with elastin in normal and fibrotic lung.

In summary, whilst in human disease and animal models up- or down-regulation of clusterin associates with diminution or enhancement of fibrogenesis respectively^11–14^ the functional significance of these changes were uncertain. Our results suggest that reduced levels of clusterin in IPF-BALF^11^ are, at least partly, due to a combination of TGF-β_1_-mediated down-regulation of fibroblast synthesis and increased binding to elastin. Functionally, clusterin promotes fibroblast proliferation and survival but does not affect differentiation or collagen synthesis/deposition. This suggests that the down-regulation of clusterin in IPF fibroblasts may be a physiologically appropriate, but insufficient, response of these cells intended to limit the development of an environment favoring unopposed fibroproliferation. Conversely the binding of clusterin to elastin in normal lung suggests that it acts as a protective extracellular chaperone facilitating the appropriate processing and maturation of elastic fibers. Although increased staining for clusterin associated with elastin was observed in fibrotic lung it is possible that together with the down-regulated expression of clusterin this was inadequate to protect the newly synthesized elastin molecules leading to their precipitation, inappropriate deposition and degradation, contributing to the disordered fibrotic extracellular matrix.

## Methods

### Patient Population

Fibrotic lung tissue was obtained from patients undergoing surgical lung biopsy or transplant surgery (IPF n = 7, aged 62 ± 4 years, four male; SSc n = 7, aged 52 ± 2 years, one male). Control lung tissue was obtained from histologically normal areas of peripheral lung removed at lung cancer resection (n = 6, aged 59 ± 7 years, two male). All tissue was obtained with appropriate informed consent and its use approved by the East Midlands – Nottingham 2 NRES Committee, Ref. 12/EM/0058. All experiments were conducted in accord with the terms of the informed consents and in accordance with relevant guidelines and regulations.

### Immunohistochemistry

Immunohistochemical staining was performed on formalin-fixed, paraffin embedded 3 μm sections of human lung tissue essentially as described previously^60^. Briefly, following dewaxing, sections were rehydrated and antigen retrieval achieved by either proteinase K digestion (20 µg/ml) for 5 minutes at room temperature (RT) for TGF-β_1_ staining or by microwaving/steaming in 10 mM citrate buffer (pH 6) for 20 minutes for clusterin or α-SMA staining. Sections were washed in TBS and endogenous peroxidase blocked with 3% hydrogen peroxide (Sigma-Aldrich) for 30 minutes at RT. After another wash sections were incubated with 2.5% (v/v) horse serum (ImmPRESS, Vector Labs CA) for 20 min at RT. Excess serum was removed and the sections incubated overnight at 4 °C with primary antibodies at pre-optimized concentrations: TGF-β_1_ (1 μg/ml, sc-146, Santa Cruz Biotechnology), clusterin (0.67 μg/ml, H330 Santa Cruz Biotechnology) and α-SMA (142 ng/ml, M0851, Dako, Denmark). Isotype IgG, in place of primary antibody, was used as a negative control. Sections were washed and incubated with anti-rabbit or anti-mouse Ig reagent as appropriate (ImmPRESS, Vector Labs, CA) for 30 minutes at RT. Further washes in TBS were performed and antibody binding was visualized using Vector NovaRED substrate (Vector Labs Pty) at established times for colour development. Sections were washed in distilled water, counterstained with Mayer’s haematoxylin, differentiated in acid alcohol (1% HCL in 70% Ethanol (v/v) in distilled water), washed in tap water, dehydrated, cleared in xylene and mounted. To visualize elastic fibers, sections were stained with the Elastic Stain Kit according to the manufacturer’s instructions (Sigma-Aldrich, UK). Section scans were performed with NanoZoomer Digital Scanner and analysis software NDP.view2 (Hamamatsu Corp).

### Fibroblast culture

Primary human lung fibroblasts were isolated and cultured from fibrotic and control lung as previously described^32,61^. Once established, cells were cultured in 175 cm^2^ tissue culture flasks (Corning, UK) in Dulbecco’s modified Eagle’s medium (DMEM) containing 10% FBS with 50 units/ml penicillin and 50 μg/ml streptomycin at 37 °C in a humidified atmosphere of air containing 10% CO_2_. Fibroblasts were passaged on reaching confluence approximately every 5–7 days and were used for experiments between passage 3 and 12. All tissue culture media and supplements were purchased from Gibco, Life Technologies, UK.

### Lentiviral shRNA induced transduction and clusterin silencing

Lentiviral plasmids with short hairpin sequences targeting clusterin were obtained from GE Healthcare UK. The target sequence providing the greatest clusterin knockdown 5′-TGAATTTCCTTATTGACGT-3′ (Oligo ID: V3LHS_337304) was selected for further analysis. As negative control, a non-targeting plasmid with sequence 5′-TCTCGCTTGGGCGAGAGTAAG-3′ was provided by GE Healthcare UK. Transduction efficiency was visualized via Turbo - GFP tag, marking cells expressing shRNA. For preparation of lentiviral particles, HEK 293 T cells at 70% confluence were co-transfected with 1.5 µg lentiviral construct (pGIPZ) plus 1 µg encapsidation plasmid (p8.91) and 1 µg envelope plasmid (pMDG) per dish using FuGENE^®^ transfection reagent as per suppliers instructions (Promega, UK) overnight at 37 °C in a humidified atmosphere of air containing 10% CO_2_. Medium was changed 18 hours after transfection and virus-containing supernatant was harvested 24, 48 and 72 hours after medium change and filtered through a 0.22 µm syringe filter unit to remove cells debris. Unconcentrated virus particles were stored at 4 °C prior to transduction of fibroblasts. Once fibroblasts reached 80% confluence the supernatant was replaced with 5 MOI (multiplicity of infection) lentiviral particles per T75 flask together with polybrene (10 µg/ml, Millipore UK Ltd.) for 6 hours at 37 °C. The supernatant was removed and replaced with 10% FBS (v/v) in DMEM. Transduction efficiency was determined 72 hours after transduction by assessing the proportion of GFP-positive cells. Thereafter, transduced cells were positively selected via resistance to puromycin (3 µg/ml, Sigma, UK) in the culture medium.

### Real-time quantitative PCR and microarray analysis

Total RNA was extracted from cultured cell monolayers using TRI-Reagent (Sigma-Aldrich), DNase treated (PrimerDesign, UK). Expression analysis was performed via microarray analysis with Illumina Infinium HumanHT-12 v4 Expression BeadChips according to supplier’s instructions by Cambridge Genomic Services (CGS, UK) or via real-time quantitative PCR (qPCR). For qPCR analysis cDNA was synthesized from 1 µg RNA using qScript cDNA SuperMix (Quanta BioSciences, USA). qPCR was performed using MESA FAST qPCR MasterMix Plus dTTP for SYBR^®^ Assay (Eurogentec, UK) or Power SYBR^®^ Green PCR Master Mix (ThermoFisher Scientific, UK) on an Eppendorf Realplex Mastercycler. Cycling conditions were as follows: 95 °C for 10 minutes; and 40 cycles of 95 °C (15 s) and 60 °C (45 s). Forward and reverse primers: *CLU* 5′-CAAGTGCCGGGAGATCTTGT-3′ (forward) and 5′-GTCAACCTCTCAGCGACCTG-3′ (reverse); *ACTA2* 5′-AATCCTGTGA-AGCAGCTCCAG-3′ (forward) and 5′-TTACAGAGCCCAGAGCCATTG-3′ (reverse); *COL1A1* 5′-ATGTAGGCCACGCTGTTCTT-3′ (forward) and 5′-GAGAGCATGAC-CGATGGATT-3′ (reverse). B2M, CYC1 and ATP5B were used as housekeeping genes.

### Analysis of fibroblast collagen deposition and clusterin expression

Fibroblast collagen deposition was assessed *in vitro* using molecular crowding conditions as described previously^62^. Briefly, cells were seeded at 1 × 10^4^ cells/well of a 96 well plate and allowed to adhere overnight. Thereafter, cells were serum deprived (0.4% FBS in DMEM) for 16 hours and neutral mixed ficoll (70 kDa ficoll at 37.5 mg/ml^−1^ and 400 kDa 25 mg/ml^−1^), L-ascorbic acid (16.6 µg/ml, Sigma-Aldrich UK) were then added to the culture medium with or without porcine TGF-β_1_ (1 ng/ml, R&D Systems). After 20–72 hours, the medium was removed and the cells fixed with ice-cold methanol. The cells were washed three times with phosphate-buffered saline (PBS), permeabilized with 0.1% Triton-X (Sigma Aldrich, UK) and blocked with 1% bovine serum albumin (Merck Milipore UK) and 3% goat serum (Sigma Aldrich, UK) in PBS for 30 min at RT. Separate wells of cells were incubated overnight with monoclonal mouse antibodies against collagen type I (Sigma, Aldrich, C2456 at 4.7 µg/ml) or mouse monoclonal against clusterin-α (Santa Cruz, sc-5289, at 2.0 µg/ml) and then washed three times with 0.05% (v/v) Tween in PBS (PBS-T). Secondary antibody (AlexaFluor 555 goat anti-mouse: A-21422, Thermo Fisher Scientific, UK) together with 1.43 nM 4,6- diamidino-2-phenylindoldilactate (DAPI, ThermoFisher Scientific, UK) were added and incubated for 1.5 hours at RT followed by three washes with 0.05% PBS-T. Fluorescently labelled proteins were visualised using an ImageXpress Micro XLS Widefield High Content Screening System and 6 - 9 images per well were analysed using the MetaXpress High Content Image Acquisition & Analysis Software (Molecular Devices, Sunnyvale, CA, USA). An integrated Multi Wavelength Cell Scoring module was used to quantify the area of fluorescent collagen I or clusterin staining. Isotype controls at concentrations of primary antibodies were used to determine fluorescent intensity thresholds for background removal. Total cell number was assessed by nuclear staining with DAPI. Data was converted into ‘mean stain integrated intensity’ (total pixel intensity over the stained area, divided by the total number of cells). Results were compared to untreated controls for each time point.

### Western Blotting

Proteins were extracted from the cell layer and 5–20 µg of total protein subjected to non-reducing SDS-PAGE using 12.5% resolving/ 4.8% stacking polyacrylamide gels. Electrophoresed proteins were electroblotted onto polyvinyldene difluoride (PVDF) membrane and immunodetection was carried out in Tris-buffered saline Tween-20 pH 7.6 (10 nM Tris, 150 nM NaCl/0.1% v/v Tween 20) with 5% w/v non-fat milk. Polyclonal rabbit or mouse-monoclonal anti-human clusterin (0.4 µg/ml, sc-8354 (discontinued) or sc-5289, Santa Cruz) or mouse monoclonal anti-human α-smooth muscle actin (7.1 ng/ml, M0851, Dako, Denmark) and goat polyclonal anti-human vinculin (0.4 µg/ml, sc-7649, Santa Cruz) antibodies were incubated overnight at RT. Secondary antibodies conjugated to horseradish peroxidase (HRP) (goat anti-rabbit, 50 ng/ml; rabbit anti-mouse, 260 ng/ml; rabbit anti-goat, 110 ng/ml, Dako, Denmark) were applied for 1.5 hours at RT. Signal detection via chemiluminescence (Luminata Crescendo Western HRP substrate, Millipore) was captured via ImageQuant^TM^ and acquisition tool ImageQuant TL 1D v.8.1 analysis software (GE healthcare, UK). Settings: Exposure type – “Precision”, exposure time −10 seconds, high sensitivity/ resolution. Protein sizes were analyzed via PageRuler Pre-stained Protein Ladder (Thermo Fisher Scientific, UK).

### Measurement of Clusterin

To measure clusterin expression, Proteome Profiler Human Apoptosis Arrays (R&D Systems, Abingdon, UK) were used in accordance with the manufacturer’s instructions. Briefly, control and fibrotic lung fibroblasts were cultured for 24 hours in serum-free medium before washing twice with ice-cold PBS and lysed using the lysis buffer provided by the supplier. The lysates were scraped from the culture plates and collected. Cell debris was removed via centrifugation (1.4 × 10^4^ g for 5 min at 4 °C) and DNA was sheared passing the lysate through a 25-gauge needle repeatedly using a 1 ml syringe. The supplied nitrocellulose membrane, pre-spotted with capture antibodies was blocked according to the supplier’s instructions, then 100 µg of total protein from the lysate was diluted with array buffer and incubated overnight with the membrane at 4 °C. Next day the membrane was washed three times to remove unbound proteins and biotinylated detection antibody was applied for 1 hour at RT followed by an additional three washes and incubation with Streptavidin-HRP for 30 minutes at RT. The membrane was washed and protein-binding detected via ECL-chemiluminescence (GE Healthcare, Amersham, UK). Secretory clusterin in fibroblast culture medium was measured by enzymeimmunoassay, according to manufacturer’s instructions (R&D, Systems Europe Ltd.).

### Transwell migration assay

Transwell migration assays were performed as previously described^63^. Primary human lung fibroblasts were seeded at 5 × 10^4^ cells in 0.4% FBS in DMEM into culture inserts (6.5 mm polycarbonate membrane pore size 8 µm, Corning Inc., NY) and allowed to migrate for 18 hours towards diffusing gradients of PDGF-BB (25 ng/ml, R&D Systems), TGF-β_1_ (1 ng/ml, R&D Systems) or human plasma-derived clusterin (1 μg/ml, Biovendor, Germany) in the lower chamber. Cells were fixed with methanol and non-migrating cells in the upper chamber were removed with a cotton swab, whilst migrated cells adhering to the lower surface of the membrane were stained with crystal violet (0.5% v/v in 10% methanol in PBS, Sigma Aldrich, Germany). Quantification of migrated cells was achieved by elution of crystal violet with 10% (v/v) glacial acetic acid and spectrophotometric analysis of absorbance at 570 nm.

### Assessment of fibroblast proliferation

Fibroblasts were seeded at a density of 4 × 10^3^ in 96 - well plates in 0.4% FBS in DMEM. 18 hours after seeding mediators were added at indicated concentrations. After a further 48–72 hours the medium was removed and the cells fixed with ice-cold methanol. The cells were washed three times with PBS, permeabilized with 0.1% Triton-X (Sigma Aldrich, UK) and blocked with 1% bovine serum albumin (Merck Milipore UK) and 3% goat serum (Sigma Aldrich, UK) in PBS for 30 minutes at RT. 1.43 nM DAPI (ThermoFisher Scientific, UK) in PBS was added and incubated for 1.5 hours at RT followed by three washes with 0.05% PBS-T. Proliferation was quantified counting numbers of DAPI-positive nuclei via ImageXpress Micro XLS Widefield High Content Screening System as described above. Changes in cell number were expressed as a percent relative to untreated control.

### Induction and detection of apoptosis

Fibroblasts were seeded at 8.0 × 10^3^ cells/well in 96 well plates and were grown to 80% confluence in DMEM supplemented with 10% FBS. Medium and unattached cells were removed and replaced with medium containing FasL (Calbiochem, CA; 0–200 ng/ml) in 5% FBS for 19 hours with or without human plasma-derived clusterin (Biovendor, Germany) at concentrations and times indicated in the figure legends. Apoptosis was detected by Alexa Fluor^®^ 647 Annexin V (BioLegend) /DAPI staining and cytometric analysis was performed via BD FACS Verse, BD FACSuite and FlowJo V10 analysis software. Verification of results was performed by morphological assessment of cell nuclei^32^. Briefly, adherent cells in 96 well plates were fixed in 70% ethanol. Cell nuclei were then stained with DAPI in PBS and analyzed by fluorescent microscopy. For each well apoptotic and non-apoptotic cells were counted in six consecutive high power fields. Cells were considered to be apoptotic if nuclei were condensed with fragmented or aggregated DNA (see Supplementary Fig. e2).

### Statistics

Data are presented as means ± SEM. Statistical evaluations were performed by ANOVA and Tukey-Kramer post hoc test for multiple comparisons or unpaired t-tests for single comparisons using GraphPad Prism version 6.0 for Mac OS X (GraphPad Software, San Diego, CA). Non-parametric data were analyzed using a Mann-Whitney U test. Two-sided p-values of less than 0.05 were considered significant.

### Data availability

The datasets generated during and/or analysed during the current study are available from the corresponding author on reasonable request.

## Electronic supplementary material

Supplementary Information and figures
