# Supplementary material for: Diverse functions of clusterin promote and protect against the development of pulmonary fibrosis
Source: Sci Rep. 2018 Jan 30;8:1906. doi: 10.1038/s41598-018-20316-1 (PMC5789849; doi:10.1038/s41598-018-20316-1)
Supplement: Supplementary file 1 — Supplementary Information and figures [file 41598_2018_20316_MOESM1_ESM.pdf]

Online Data Supplement

Title: **Diverse functions of clusterin promote and protect against the development of pulmonary fibrosis.**

Lizzy Peix[1, 2], Iona C. Evans[1, 3], David R. Pearce[1], Juliet K. Simpson[2], Toby M. Maher[4, 5], Robin J. McAnulty[1*]

[1]UCL Respiratory Centre for Inflammation and Tissue Repair, Rayne Building, University College London, London, WC1E 6JF, UK.

[2]Current address: GlaxoSmithKline, Stevenage, UK.

[3]Current address: UCL Institute for Woman's Health, University College London, London, UK.

[4]NIHR Respiratory Biomedical Research Unit, Royal Brompton Hospital, London, UK.

[5]Fibrosis Research Group, National Heart and Lung Institute, Imperial College, London, UK.

Online supplement figures

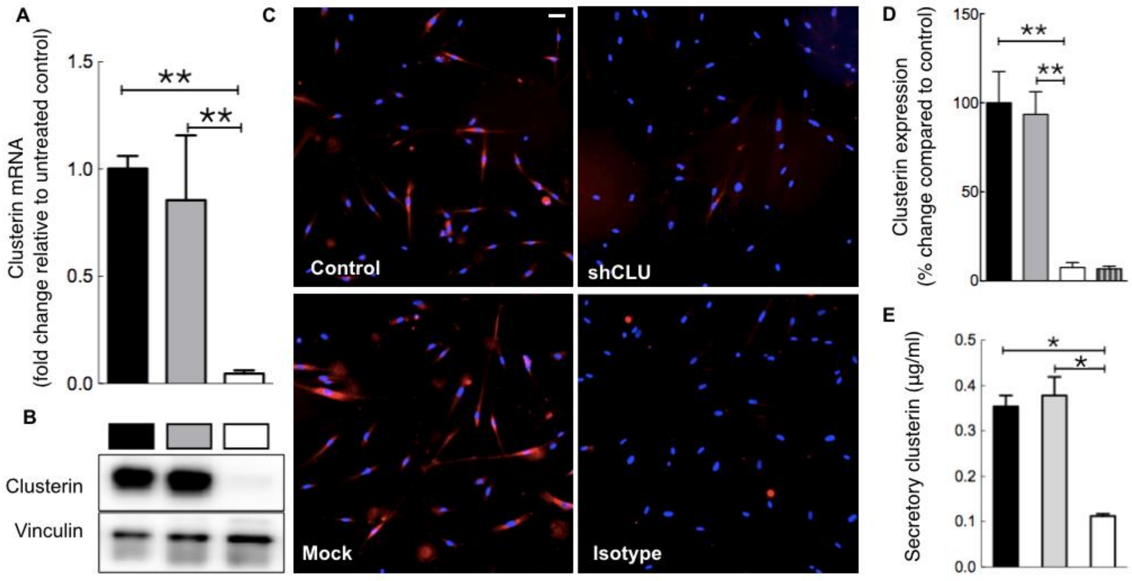

Figure e1

Figure e1. shRNA - induced knockdown of clusterin in human normal lung fibroblasts. Clusterin gene and protein expression was silenced post lentivial transfection with shRNA targeting the clusterin gene (shCLU, open bars) compared with non-silencing (mock, grey bars) and non - transduced fibroblasts (control, black bars). qPCR (A, n=3), western blot (B), immunofluorescence staining (panel C, clusterin red, nuclei - blue, n=3, quantitative analysis in D, isotype striped bar) confirmed low CLU gene and protein expression in shCLU transfected fibroblasts. ELISA analysis shows reduced levels of secretory clusterin post shRNA transduction (n = 2). Scale bar in panel C represents 10 µm.

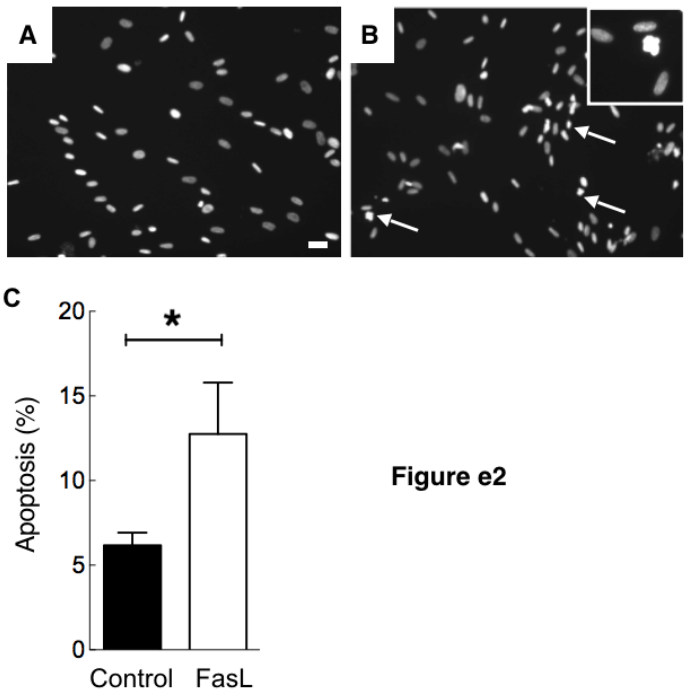

Figure e2

Figure e2 Microscopic assessment of FasL-induced apoptosis in control lung fibroblasts. Apoptosis was assessed morphologically in fixed, untreated cells that were permeabilized followed by nuclei staining with DAPI: (A) untreated fibroblasts, (B) FasL-treated fibroblasts. Apoptotic nuclei initially demonstrate condensation of chromatin (B, high-power inset) before progressing to form dense, highly fluorescent apoptotic bodies (arrows in B). (C) Quantitative analysis was determined by counting apoptotic and nonapoptotic cells in four high-power fields for each experimental replicate (n=6). Scale bar in A represents 10 µm.

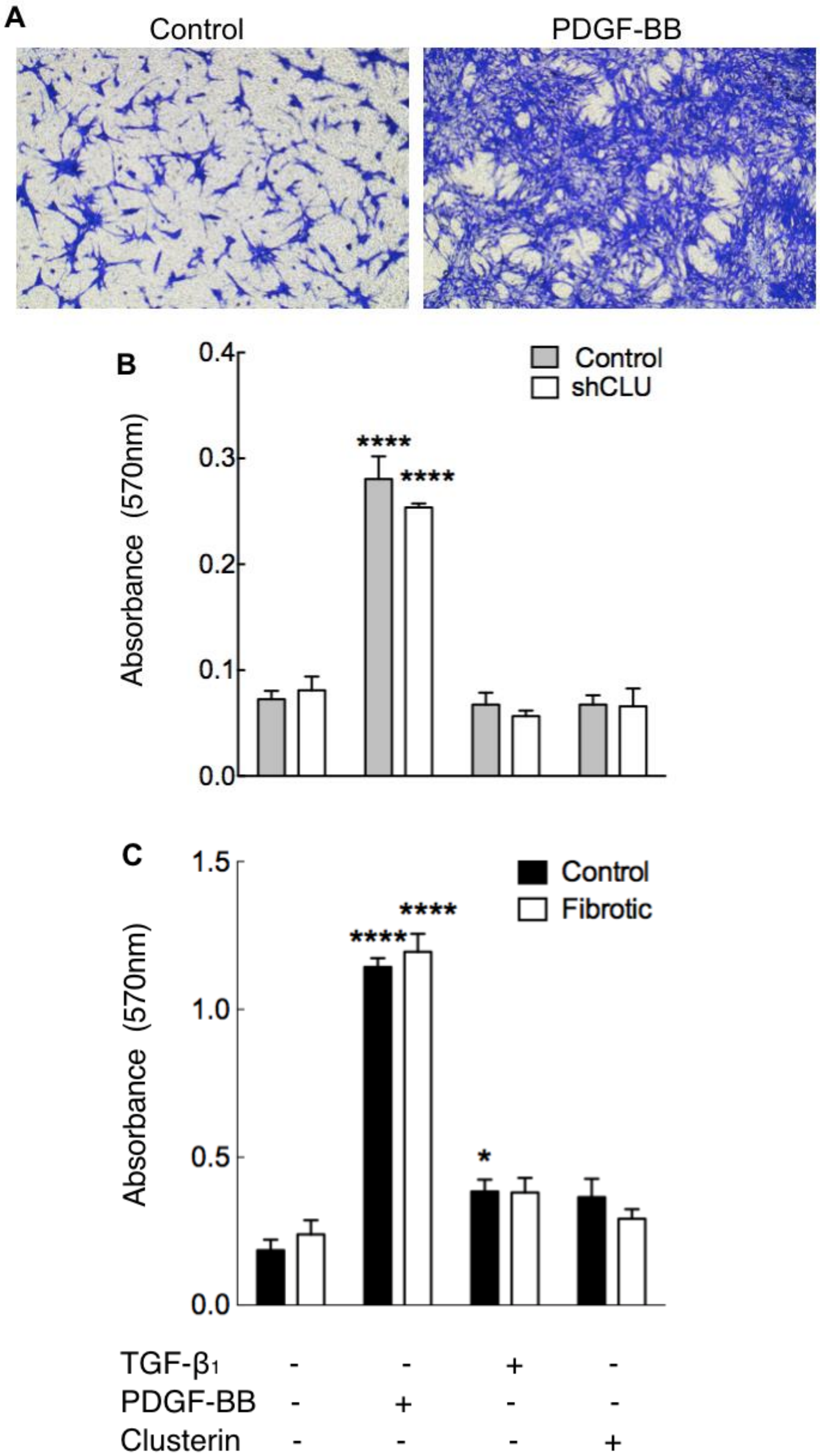
A
Control
PDGF-BB
B
Control
shCLU
Absorbance (570nm)
0.4
0.3
0.2
0.1
0.0
****
****
C
Control
Fibrotic
Absorbance (570nm)
1.5
1.0
0.5
0.0
****
****
*
TGF-β1 - - + -
PDGF-BB - + - -
Clusterin - - - +

Figure e3

Figure e3 High exogenous clusterin and low intracellular clusterin do not affect lung fibroblast migration in control and fibrotic fibroblasts. (A) Representative bright field images of crystal violet-stained migratory fibroblasts on transwell polycarbonate membranes, showing a three fold increase in migration in response to PDGF-BB (25 ng/ml) compared with unstimulated controls. Since migratory responses of mock-transduced and untransduced fibroblasts did not show significant differences, migratory responses of clusterin deficient fibroblasts were compared to mock-transduced controls only. (B-C) Comparison of migratory response in mock-transduced (control) compared with shCLU deficient fibroblasts (B) and control fibroblasts with fibrotic lung fibroblasts (C) in response to no stimuli or PDGF-BB (25 ng/ml), TGF-β_1 (1 ng/ml) and exogenous, human plasma derived clusterin (10 µg/ml). Quantification of migrated cells via crystal violet solubilization and spectrophotometric analysis of the absorbance at 570 nm. Data represent mean ± SEM of two independent experiments. *$P < 0.05$, ****$P < 0.0001$ compared with respective control.

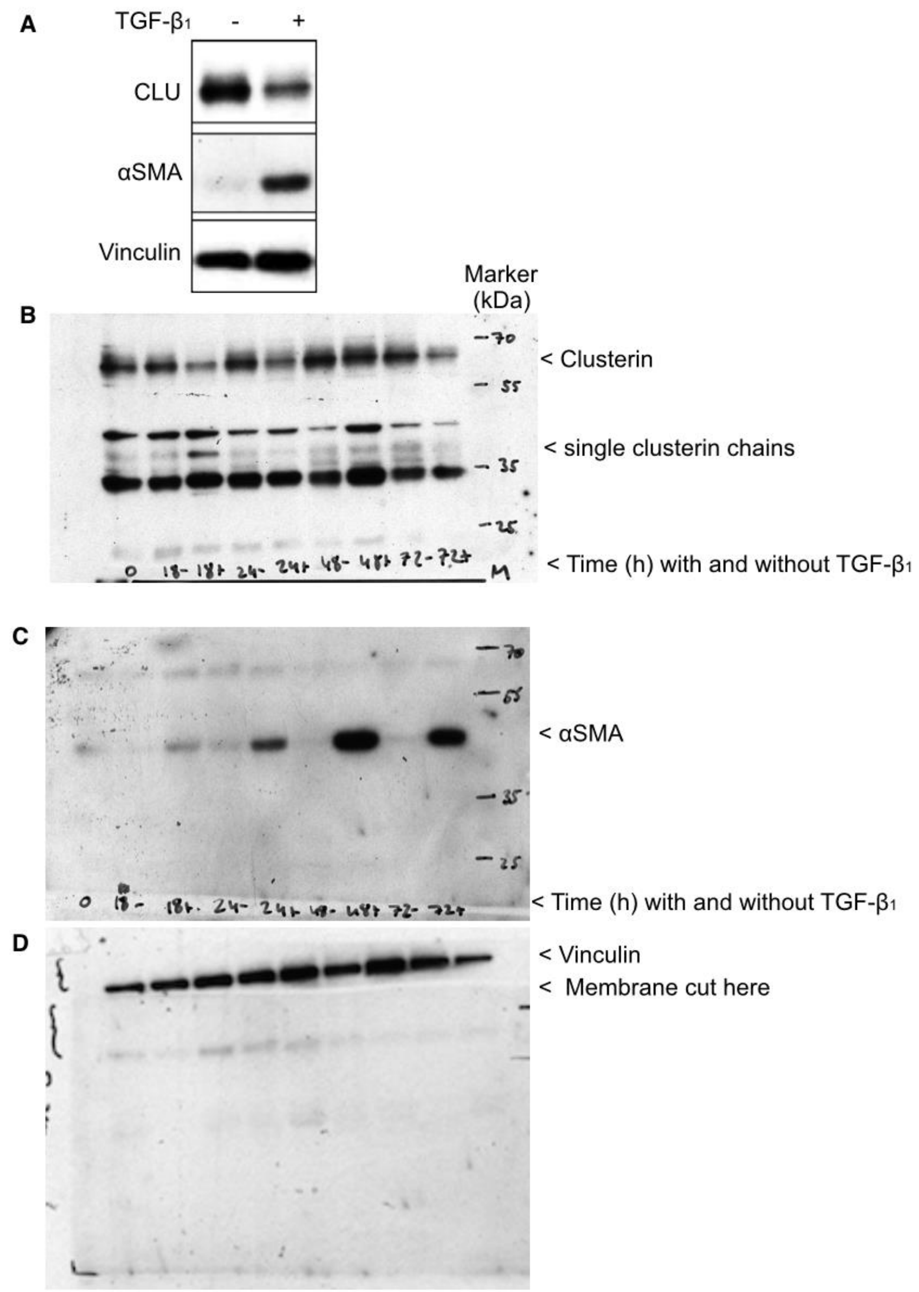

Figure e4 Presentation of full-length blot for cropped blot in Figure 4D.

(A) Cropped blot, different parts of the same gel presented in Figure 4D. (B) Blot probed with anti-clusterin antibody (0.4 µg/ml, sc-8354, Santa Cruz). (C) After stripping, the bottom of the membrane was reprobed with anti-αSMA (7.1 ng/ml, M0851, Dako, Denmark) and the top of the membrane (D) was reprobed with anti-Vinculin antibody (0.4 µg/ml, sc-7649, Santa Cruz). Molecular Marker bands as indicated on the right hand side of the blots.

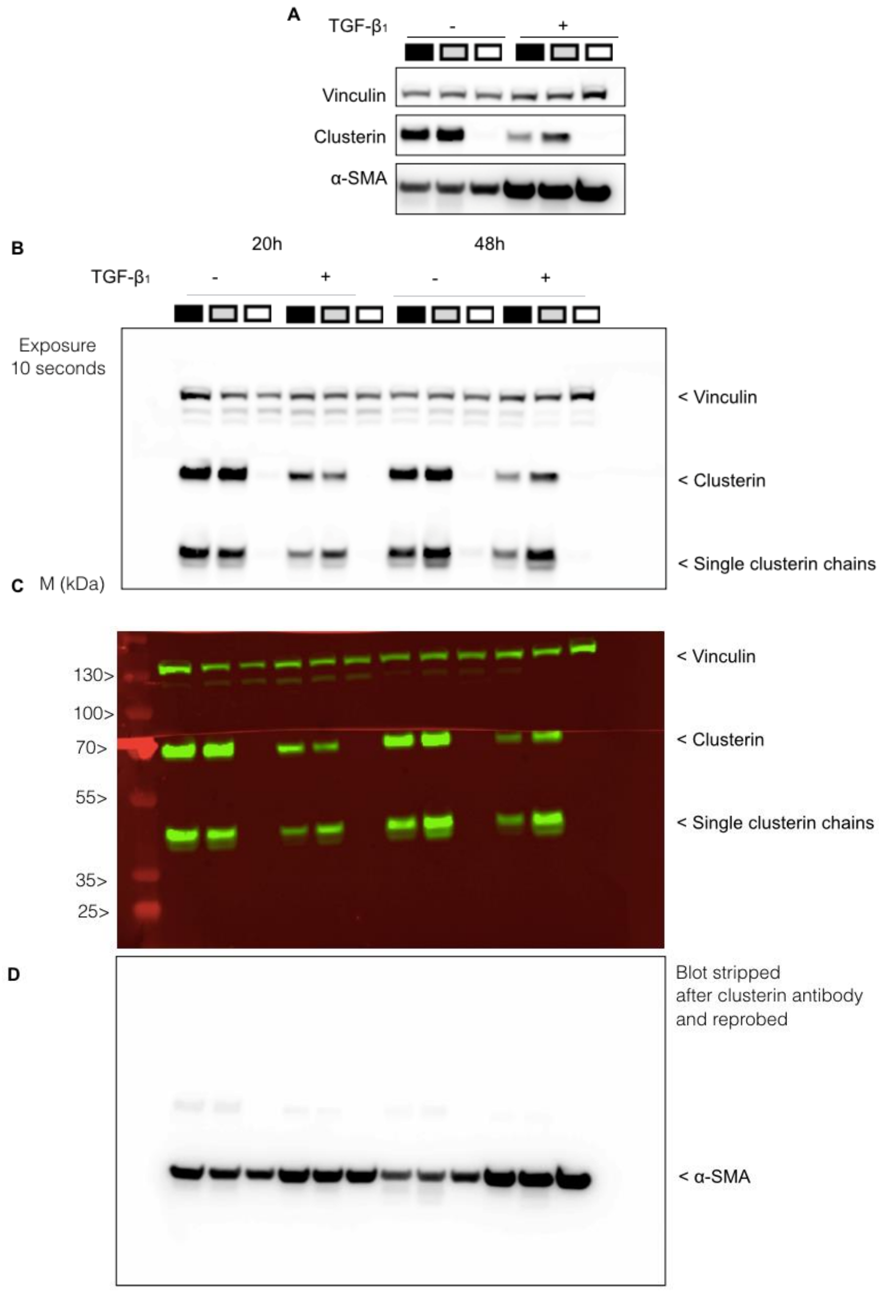

Figure e5 Presentation of full-length blot for cropped blot in Figure 5B.

(A) Cropped blot, different parts of the same gel presented in Figure 5B. (B) After cutting the membrane the bottom of the blot was probed with anti-clusterin antibody (0.4 µg/ml, sc-5289, Santa Cruz) and the top of the blot was probed with anti-vinculin antibody (0.4 µg/ml, sc-7649, Santa Cruz). (C) Molecular Band Marker on membrane (in kDa). (D) After stripping, the bottom of the membrane was reprobed with anti-αSMA (7.1 ng/ml, M0851, Dako, Denmark).

49
